# Sequencing Meets Repetition Coding: Quadratic Substitution Error Suppression through Homotrimer Redundancy

**DOI:** 10.34133/csbj.0271

**Published:** 2026-11-12

**Authors:** Jianfeng Sun, Adam P. Cribbs

**Affiliations:** ^1^ Thrust of Bioscience and Biomedical Engineering, Hong Kong University of Science and Technology (Guangzhou), Guangzhou 511400, China.; ^2^ Botnar Research Centre, University of Oxford, Oxford OX3 7LD, UK.

## Abstract

Homotrimer unique molecular identifiers encode each nucleotide 3 times to suppress substitution errors. Here, we derive decoding-error probabilities for this quaternary repetition code under majority voting with uniform random tie-breaking. Under independent substitutions (base error probability) p, the decoding error probability is $g\left(p\right)=\left(7/3\right)p^{2}-\left(4/3\right)p^{3}$. For nonuniform conditional substitution probabilities $\pi_{i}$, it becomes $g\left(p,\pi \right)=\left(1-p\right)p^{2}\left(2+\sum_{i=1}^{3}\pi_{i}^{2}\right)+p^{3}$. Exhaustive enumeration and Monte Carlo simulations verify these results. This analysis quantifies the error suppression attributable to homotrimer redundancy under a specified local decoder, without modeling correlated errors, indels, or downstream molecular-counting accuracy.

## Introduction

Unique molecular identifiers (UMIs) enable molecular quantification, but nucleotide substitution errors can generate spurious UMI sequences and compromise counting accuracy [1]. Recently introduced homotrimer UMIs, originally inspired by triple modular redundancy (TMR) that leverages a fault-tolerance principle to exploit 3-fold repetition for error suppression through majority voting, have demonstrated their experimental utility, with set-cover-based correction subsequently explored as a complementary computational strategy [2]. However, such downstream correction methods are conceptually distinct from the intrinsic error-suppression mechanism of homotrimer redundancy, which remains insufficiently characterized mathematically, particularly under elevated substitution-error rates.

In communication theory, TMR is closely related to length-3 repetition codes [3]. Unlike binary codes, homotrimer UMIs operate over 4 nucleotides, where substitutions can produce 3 different bases without a majority. This difference requires additional consideration of ambiguous decoding outcomes and substitution probabilities.

Here, we provide a simple probabilistic derivation to quantify error suppression by homotrimer redundancy. Assuming independent substitutions, we derive the decoding-error probability under majority voting with uniform random tie-breaking, considering both equal and unequal substitution probabilities. We verify the results through computational simulations and examine the extent and limitations of quadratic error suppression, particularly at elevated error rates. This analysis focuses on the intrinsic robustness of homotrimer redundancy rather than downstream correction algorithms or overall molecular-quantification performance.

## Methods

### TMR and homotrimer UMIs

TMR is a fault-tolerant error correction technique in which a single data bit is copied and transmitted 3 times (e.g., 0 is encoded as 000) [4]. This technique has widely been used to protect data integrity in noisy communication channels across fields, such as aerospace systems [5] and satellite communications [6].

In sequencing, the same principle of redundancy has been echoed and applied in homotrimer UMI demultiplexing to counteract synthesis errors and then remove polymerase chain reaction (PCR) artifacts for accurate molecular quantification [2]. A homotrimer UMI consists of building blocks in which each nucleotide is tripled (e.g., A turns to AAA). This design allows errors affecting one nucleotide within a triplet to be resolved by majority voting.

### From binary repetition coding to homotrimer block error correction

In a communication process, we assume that Alice and Bob represent the sender and receiver of a message, respectively. Suppose that Alice wishes to transmit the bit 0. To protect against transmission errors, she applies TMR to encode 0 as 000, also known as the (3,1) repetition code [7]. Due to potential noise in the channel, Bob may receive 1 of 8 possible 3-bit combinations. As shown in Fig. 1A, Bob applies majority voting to decode them into the original bit. If 2 or more of the received bits agree, that value is interpreted as the intended message. In this context, if a single bit is flipped regardless of its position, the voting mechanism can correctly recover the original message 0. Upon transmission, if each bit has an independent probability p of flipping, then the probability f of a decoding error after applying majority voting is $3p^{2}\left(1-p\right)+p^{3}$, which simplifies to

$$f\left(p\right)=3p^{2}-2p^{3}$$ (1)

**Fig. 1.**
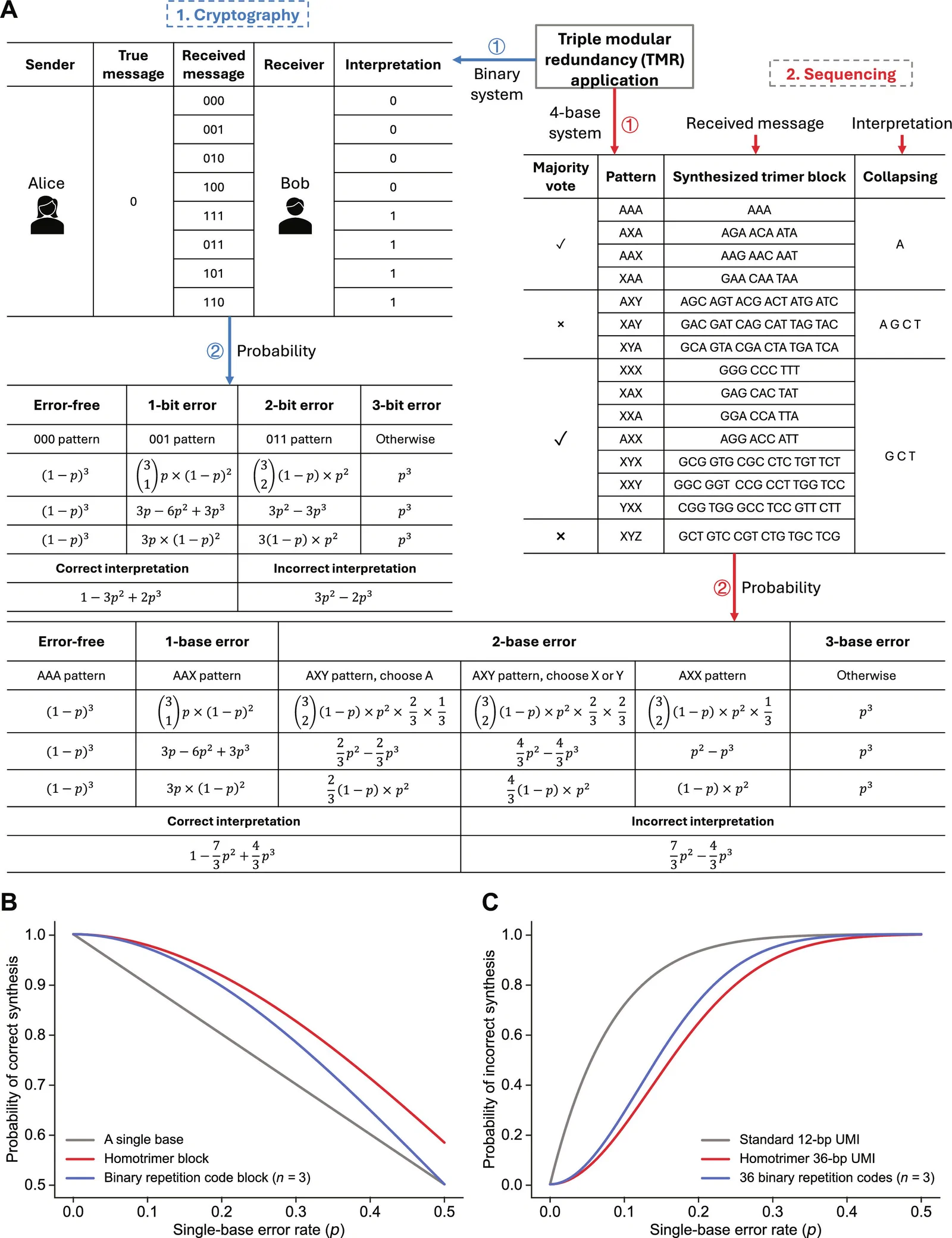
A coding-theoretic model of homotrimer unique molecular identifier (UMI) decoding. (A) Conceptual analogy between repetition-code error correction and nucleotide-level error correction in sequencing using triple modular redundancy (TMR). On the left, a classic (3,1) repetition-code scheme is illustrated in a binary system, where Alice sends a bit 0 encoded as 000, and Bob decodes received messages using majority voting. A probability model quantifies the probability of correct or incorrect interpretation based on the number and pattern of bit flips. On the right, we extend this logic to sequencing, where each base is encoded as a homotrimer (e.g., AAA). Unlike binary systems, DNA sequencing operates in a 4-base system with substitution errors (e.g., A → C, G, or T). The majority-vote decoding is retained when possible, and collapsing is performed by random selection when no majority exists (e.g., ACG). A probability model is derived to quantify correct *vs.* incorrect block interpretation as a function of per-base error rate p, accounting for the combinatorics of 1, 2, and 3 substitution error scenarios. (B) Theoretical probability of correct synthesis for a single nucleotide (gray, 1−p), a binary repetition-code block of 3 bits (royal blue, $1-f\left(p\right)$), and a homotrimer block (crimson, $1-g\left(p\right)$) as a function of single-base error rate p. The homotrimer block demonstrates resilience to substitution errors across the full range of p. (C) Theoretical probability of incorrect synthesis for a 12-bp standard UMI (gray, $1-{\left(1-p\right)}^{12}$), a 36-bit binary (3,1) repetition code (royal blue, $1-{\left(1-f\right)}^{12}$), and a 36-bp homotrimer UMI (crimson, $1-{\left(1-g\right)}^{12}$). The homotrimer UMI shows consistently lower error rates than both alternatives, underscoring the benefit of homotrimer-based redundancy in biological substitution settings.

The homotrimer block design is conceptually equivalent to the (3,1) repetition code used in binary systems. However, while errors in binary communication are typically modeled as bit flips (e.g., 0 → 1), sequencing errors are characterized by substitutions to 1 of 3 alternative nucleotides (e.g., A → C, G, or T). This fundamental difference in error structure necessitates a distinct mathematical framework to accurately model the probability of decoding errors. Unlike binary repetition codes, where a majority vote always yields a result as long as 2 bits agree, homotrimer blocks can fail to produce a majority when 2 or 3 distinct substitution errors occur within a triplet. In such cases, majority voting may not resolve the original base. Nevertheless, if the original nucleotide remains present in 1 of the 3 positions, a decoder that selects randomly among the triplet still retains a nonzero probability of recovering the correct base. This stochastic element has no analog in classical repetition coding. Consequently, an appropriate probabilistic model must incorporate both deterministic majority voting and stochastic tie-breaking scenarios to reflect the full spectrum of outcomes in substitution-driven sequencing errors. Therefore, the probability g of a mistakenly voted nucleotide from a homotrimer block is calculated as

$$g\left(p\right)=\frac{7}{3}p^{2}-\frac{4}{3}p^{3}$$ (2)

### Local decoder and channel assumptions

Each intended nucleotide X is encoded as the physical triplet XXX. Conditional on X, the 3 observed positions undergo independent substitutions with the same probability p. In the symmetric model, an incorrect position contains each alternative nucleotide with conditional probability 1/3. This is a quaternary symmetric channel [8]. The decoder returns the base present at least twice. If all 3 observed bases differ, it chooses uniformly among those 3 bases independently for each tied block. It uses no quality scores, other reads, abundance information, or set-cover constraints. Trimer boundaries are assumed known and preserved. Insertions and deletions are excluded from the current study.

With zero or one substitution, decoding is correct. Exactly 2 substitutions occur with probability $3\left(1-p\right)p^{2}$. Under symmetric substitutions, the 2 incorrect bases coincide with probability 1/3, causing an incorrect majority. Otherwise, the triplet contains the intended base and 2 different alternatives, so uniform tie-breaking is incorrect with probability 2/3. With 3 substitutions, the intended base is absent and decoding is always incorrect.

### Nonuniform substitutions and *q*-ary channels

The coefficient 7/3 depends on the substitution spectrum. Let $\pi_{1}$, $\pi_{2}$, and $\pi_{3}$ be the conditional probabilities of the 3 incorrect bases for a given intended nucleotide, with $\pi_{1}+\pi_{2}+\pi_{3}=1$, and define $S_{2}=\pi_{1}^{2}+\pi_{2}^{2}+\pi_{3}^{2}$. Retaining independent, identically distributed positions and the same decoder, 2 incorrect bases agree with probability $S_{2}$. Thus, the exact error probability is

$$g\left(p,\pi \right)=\left(1-p\right)p^{2}\left(2+S_{2}\right)+p^{3}$$ (3)

Since $1/3\leq S_{2}\leq 1$, the coefficient of the leading quadratic term ranges from 7/3 for uniform alternatives to 3 when errors select only one alternative. The latter case gives the binary expression in Eq. (1). Consequently, independence preserves the quadratic small-p scaling for every fixed spectrum, whereas the additional reduction relative to binary repetition depends on how substitutions are distributed.

More generally, a symmetric channel over an alphabet of size Q≥2 assigns conditional probability $1/\left(Q-1\right)$ to each incorrect symbol [3]. For the same length-3 decoder,

$$g_{Q}\left(p\right)=\left(2+\frac{1}{Q-1}\right)\left(1-p\right)p^{2}+p^{3}$$ (4)

Setting Q=2 recovers Eq. (1), and Q=4 recovers Eq. (2). Three distinct observations can occur for alphabets larger than 2, so random tie-breaking is a conventional possibility in *q*-ary repetition decoding; it is absent for an odd-length binary repetition code.

### Analysis of block error probabilities

We plotted the correct-decoding probabilities of 1−p, $1-f\left(p\right)$, and $1-g\left(p\right)$ over the illustrative range 0≤p≤0.5 for correct UMI synthesis with a resolution of 500 steps. As shown in Fig. 1B, the homotrimer block can successfully be synthesized with a larger probability than the (3,1) repetition code under the classical binary bit-flipped model f. The landscapes of both error probability distributions demonstrate a more pronounced improvement in mitigating error profiles than the use of a sole nucleotide.

The principal protection at low error rates is the change from an unprotected error probability of order p to a decoded-symbol error probability of order $p^{2}$. For symmetric substitutions, the ratio $g\left(p\right)/f\left(p\right)$ approaches 7/9 as p approaches zero, i.e., the quaternary model having a 22.2% lower leading coefficient than the binary model. This coefficient comparison is separate from the much larger change in error order.

### Analysis of full-length UMI error probabilities

For a UMI with L independent blocks and the same decoded-symbol error probability g in each block, the probability that at least one decoded symbol differs from the intended UMI is

$$E_{L}=1-{\left(1-g\right)}^{L}$$ (5)

To place our analysis in a realistic context, we focused on the 10x Genomics Chromium Single Cell 3′ v3 chemistry. It uses a 12-bp UMI (i.e., L=12), which we referred to as the conventional monomer UMI [1]. As shown in Fig. 1C, the probability of incorrect synthesis of a monomer UMI against per-base error rate p is delineated by a gray line, where any single nucleotide error results in failed synthesis of a UMI. In contrast, the homotrimer UMI (36-bp) design substantially reduces the probability of decoding errors compared to the standard monomer UMI, where each triplet is decoded by majority voting or by random selection when no majority exists. In this setting, only erroneous decoding of any triplet contributes to failure. Importantly, the probability distribution of incorrect synthesis of homotrimer UMIs demonstrates greater error tolerance compared to the classical bit-flip error model, highlighting the advantages of homotrimer-based redundancy in the biological substitution setting.

It also illustrates that when L=12, g is replaced by p, $f\left(p\right)$, or $g\left(p\right)$ for an unprotected sequence, binary repetition, or homotrimer decoding, respectively. A 12-bp monomer UMI and a 36-bp homotrimer UMI encode the same 12 quaternary source symbols and have up to $4^{12}=2^{24}$ possible identifiers. They therefore share logical diversity but differ 3-fold in physical length. The 12-block binary example carries only 12 source bits, with 2^12^ possible messages and 36 transmitted bits. It illustrates alphabet dependence and is not an information-equivalent system comparison. Equation (5) concerns exact barcode-sequence identity, not downstream molecular-counting accuracy.

## Results

### Illustrative error probabilities

Although DNA polymerase substitution error rates vary across the literature, we adopted a representative substitution error rate of 10^−6^, based on empirical measurements from a benchmark study [9] to estimate the probability of incorrect decoding of a homotrimer block. Substituting into our model yields

$$g\left(10^{-6}\right)=\frac{7}{3}10^{-12}-\frac{4}{3}10^{-18}\approx 2.33\times 10^{-12}$$ (6)

We extended this analysis to additional substitution error rates, including 10^−8^ and 10^−4^, to compute corresponding homotrimer block error probabilities (Table 1). Note that it gives illustrative effective substitution probabilities rather than polymerase-specific predictions. Across this range, the probability of decoding error scaled approximately with the square of the per-base error rate (multiplied by about 2.33), indicating robust suppression of substitution errors via the homotrimer encoding and majority-voting strategies.

**Table 1.** Example error rates per nucleotide, homotrimer block, and binary (3,1) repetition code

DNA polymerase error rate	Homotrimer block error rate	Binary (3,1) repetition-code error rate
10^−8^	2.33 × 10^−16^	3.00 × 10^−16^
10^−6^	2.33 × 10^−12^	3.00 × 10^−12^
10^−4^	2.33 × 10^−8^	3.00 × 10^−8^
10^−2^	2.32 × 10^−4^	2.98 × 10^−4^

Moreover, for a distinct base-calling-only illustration, a calibrated Phred score of 30 corresponds to a base-call error probability of 10^−3^ [10]. If these errors are assumed to consist solely of independent, uniformly distributed substitutions and upstream errors are absent, Eq. (2) gives $g\left(10^{-3}\right)=2.332\times 10^{-6}$. This conditional illustration does not assign an empirical error rate to a sequencing platform or include PCR and synthesis errors.

### Computational validation

We verified the formula independently by enumerating all 64 ordered nucleotide triplets at 101 values of p for each of 3 synthetic conditional spectra: (1/3,1/3,1/3), (0.8,0.1,0.1), and (1,0,0). The maximum absolute difference from Eq. (3) was below 1.34 × 10^−15^. We also generated 2,000,000 independent triplets per spectrum at p=0.01, 0.05, 0.10, and 0.25, explicitly applying majority voting and random tie-breaking, for 24,000,000 simulated triplets in total. Estimates differed from analytical predictions by at most 2.21 binomial standard errors. Pointwise 95% Wilson intervals and all numerical results are reported in Fig. S1 and Table S1. These calculations verify the derivation and implementation under the assumed channel, not the biological validity of its assumptions.

## Discussion

Homotrimer UMIs can be understood as length-3 quaternary repetition codes. The present calculation isolates the local majority-vote decoder with uniform random tie-breaking and shows how its substitution-error coefficient depends on the conditional error spectrum. In the previous workflow, inconclusive observations can instead be resolved jointly across UMIs within a gene and, for single-cell data, within a cell using set-cover optimization in which set coverage was compared with majority voting [2]. Such decisions depend on the observed collection of reads and cannot be inferred from the single-block probability derived here. Quality-score-informed decoding would likewise require a different observation model and decision rule.

Independence is essential to quadratic suppression. For example, if a common event changes all 3 copies to the same incorrect base with probability p, each position has error probability p, but the decoder also fails with probability p. PCR inheritance can create dependencies between related reads, while sequence context and platform-specific effects may invalidate an independent-position description. The numerical checks do not establish independence in experimental data, and the synthetic spectra have not been calibrated to empirical substitution matrices. Indels, homopolymer-associated effects and loss of trimer framing require additional modeling.

Barcode-sequence recovery is only one component of molecular quantification. Downstream UMI clustering may recover some altered sequences [1], whereas a correctly decoded barcode does not eliminate collisions, chimeras, sampling effects, or amplification-related count distortion. Selecting a UMI length would require joint consideration of logical barcode diversity, collision risk, decoding error, and the 3-fold physical-length cost. No such optimization is presented. The analysis therefore provides a transparent reference for a specified local decoder under explicit assumptions, with empirical calibration and workflow-level evaluation remaining separate tasks.

## Data Availability

The probabilistic analysis is made as a separate module in UMIche. The Python scripts for reproducing the experiments conducted in this work are publicly accessible through https://github.com/2003100127/umiche/blob/main/umiche/plot/line/TripletErrorCode.py, https://github.com/2003100127/umiche/blob/main/umiche/deduplicate/method/trimer/Collapse.py, https://github.com/2003100127/umiche/blob/main/umiche/deduplicate/TMR.py, and https://github.com/2003100127/umiche/blob/main/umiche/homotrimer.py. In addition, a standalone package for reproducible analysis is provided as Supplementary Code. The documentation for using this module is made available publicly at https://2003100127.github.io/umiche/tmranalysis.

## Supplementary Materials

Supplementary 1 Supplementary Code Supplementary Methods Fig. S1 Table S1

## References

[1] Smith T, Heger A, Sudbery I. UMI-tools: Modeling sequencing errors in unique molecular identifiers to improve quantification accuracy. Genome Res. 2017;27(3):491–499. 10.1101/gr.209601.116 PMC5340976 28100584

[2] Sun J, Philpott M, Loi D, Li S, Monteagudo-Mesas P, Hoffman G, Robson J, Mehta N, Gamble V, Brown T Jr, et al. Correcting PCR amplification errors in unique molecular identifiers to generate accurate numbers of sequencing molecules. Nat Methods. 2024;21(3):401–405. 10.1038/s41592-024-02168-y PMC10927542 38317008

[3] Huffman WC, Pless V. *Fundamentals of error-correcting codes*. Cambridge (UK): Cambridge University Press; 2003.

[4] Lyons RE, Vanderkulk W. The use of triple-modular redundancy to improve computer reliability. IBM J Res Dev. 1962;6(2):200–209.

[5] Arifeen T, Hassan AS, Lee J-A. Approximate triple modular redundancy: A survey. IEEE Access. 2020;8:139851–139867.

[6] Wirthlin MJ, Keller AM, McCloskey C, Ridd P, Lee D, Draper J. SEU mitigation and validation of the LEON3 soft processor using triple modular redundancy for space processing. In: *Proceedings of the 2016 ACM/SIGDA international symposium on field-programmable gate arrays*. New York (NY): ACM; 2016. p. 205–214.

[7] Shimeall TJ, Spring JM. *Introduction to information security*. Boston (MA): Syngress; 2014. Chapter 14, Recognition strategies: Integrity detection; p. 303–316.

[8] Roth R. *Introduction to coding theory*. Cambridge (UK): Cambridge University Press; 2006.

[9] Cline J, Braman JC, Hogrefe HH. PCR Fidelity of Pfu DNA polymerase and other thermostable DNA polymerases. Nucleic Acids Res. 1996;24(18):3546–3551. 10.1093/nar/24.18.3546 PMC146123 8836181

[10] Ewing B, Green P. Base-calling of automated sequencer traces using Phred. II. Error probabilities. Genome Res. 1998;8(3):186–194. 9521922

