# Supplementary material for: Sequencing Meets Repetition Coding: Quadratic Substitution Error Suppression through Homotrimer Redundancy
Source: Comput Struct Biotechnol J. 2026 Nov 12;35(1):0271. doi: 10.34133/csbj.0271 (PMC13756771; doi:10.34133/csbj.0271)
Supplement: Supplementary file 1 [file csbj.0271.f1.zip › Supplementary_Material.docx]

***Supplementary Material***

**Sequencing meets repetition coding: quadratic substitution error suppression through homotrimer redundancy**

Jianfeng Sun^1,#^ and Adam P. Cribbs^2,#^

^1^ Thrust of Bioscience and Biomedical Engineering, Hong Kong University of Science and Technology (Guangzhou), Guangzhou 511400, China

^2^ Botnar Research Centre, University of Oxford, Oxford OX3 7LD, UK

Address correspondence to: [jianfengsun@hkust-gz.edu.cn](mailto:jianfengsun@hkust-gz.edu.cn); [adam.cribbs@ndorms.ox.ac.uk](mailto:adam.cribbs@ndorms.ox.ac.uk)

**Supplementary Methods**

**Conditional substitution spectra and the general formula**

For a fixed intended base $x$, an observed position equals $x$ with probability $1-p$ and equals the three incorrect bases with probabilities $p\pi_{1}$, $p\pi_{2}$ and $p\pi_{3}$. The three positions are independent with the same probabilities. For exactly two substitutions, the chance that the incorrect symbols coincide is $S_{2}=\pi_{1}^{2}+\pi_{2}^{2}+\pi_{3}^{2}$. Matching incorrect symbols force an incorrect majority; distinct incorrect symbols leave a three-way tie, which is resolved incorrectly with probability 2/3. The exact contribution is therefore

$g(p,\pi)=3(1-p)p^{2}[S_{2}+\frac{2}{3}(1-S_{2})]+p^{3}$	(S1)

$g(p,\pi)=(2+S_{2})p^{2}-(1+S_{2})p^{3}$	(S2)

Both expressions are algebraically identical to main-text Eq. (3). The bounds $1/3\leq S_{2}\leq1$ imply that the uniform-quaternary expression is the minimum and the binary expression is the maximum for this decoder at a fixed $p$. The error reduction relative to binary repetition is

$f(p)-g(p,\pi)=(1-S_{2})(1-p)p^{2}$	(S3)

**Interpretation of a future empirical substitution matrix**

If an empirical conditional matrix $M_{xy}=P(Y=y| X=x)$ is available from trusted observed-versus-intended base assignments, one can define the row-specific effective substitution probability $p_{x}=1-M_{xx}$. For $p_{x}>0$, the off-diagonal entries give $\pi_{xy}=M_{xy}/p_{x}$ for $y\neq x$. Their squared sum defines $S_{2,x}$, and Eq. (3) applies to that row if the three positions are independent and share that row distribution. When $p_{x}=0$, its decoded-symbol error is zero. Base composition would then determine how row-specific results are aggregated. Context dependence, unequal position distributions and correlations require additional modelling. This is a description of how the formula could be applied; no empirical matrix was estimated or used in this study.

**Exhaustive enumeration**

For each synthetic spectrum, the script enumerates all $4^{3}=64$ ordered observations. Each observation receives the product of its three conditional symbol probabilities. The enumeration decoder evaluates its actual majority outcome or, for three distinct symbols, the fraction of the three possible tie outcomes that are incorrect. Weighted errors are summed without invoking the closed-form expression. Checks use 101 equally spaced values of $p$ from 0 to 1 for each of the three spectra, for 303 comparisons. The maximum absolute numerical discrepancy is below $1.34\times10^{-15}$. The wider enumeration interval verifies the algebra of the fixed decoder; it does not imply that repetition improves performance throughout that interval.

**Monte Carlo simulation settings**

Each of 12 settings contains 2,000,000 independent triplets. The error probabilities are 0.01, 0.05, 0.10 and 0.25; the three conditional spectra are uniform (1/3, 1/3, 1/3), biased (0.8, 0.1, 0.1) and concentrated on one alternative (1, 0, 0). These settings are illustrative and are not estimates from a platform. The master seed is 20261027; NumPy SeedSequence generates a separate child stream for each setting, and observations are processed in batches of 200,000. The script generates each observed base from the appropriate categorical distribution, tests for a majority, and samples a uniform choice among the three positions when all observed bases differ. A trial is an error if the decoded symbol differs from the fixed intended symbol. The intended symbol is represented by 0; symbols 1, 2 and 3 denote its incorrect alternatives.

Let $k$ denote the decoded errors among $N=2,000,000$ trials and let $\hat{g}=k/N$. The reported Monte Carlo estimate is $\hat{g}$. Its standardized difference from the analytical probability is $(\hat{g}-g)/\sqrt{g(1-g)/N}$. Two-sided pointwise 95% Wilson intervals use $z=1.959963984540054$. They are not simultaneous confidence bands. The largest absolute standardized difference is 2.204436, and 10 of the 12 individual intervals contain their analytical probabilities. Both interval misses are retained in the supplied data; no seed or simulation result was selected to force coverage. This sampling comparison complements the independent exact enumeration.

**Supplementary Fig. S1.** Independent computational validation of the local decoder model. (a) Analytical error probabilities (lines) and Monte Carlo estimates (symbols) for three synthetic conditional substitution spectra. Each setting contains 2,000,000 independently generated triplets decoded by majority voting with uniform random tie-breaking. Error bars show two-sided pointwise 95% Wilson intervals. (b) Ratios of simulated to analytical probabilities; the dashed line denotes unity. Ten of 12 analytical values lie within their individual intervals, and the maximum absolute deviation is 2.21 binomial standard errors. Small horizontal offsets distinguish overlapping spectra in panel (b). These simulations test the mathematical model and its implementation under stipulated assumptions, rather than empirical sequencing-error behaviour.

**Supplementary Table S1.** Decoded-symbol errors in all Monte Carlo settings. Each row contains 2,000,000 trials.

| **Spectrum** | **p** | **Errors** | **Analytical** | **Simulated** | **95% Wilson interval** |
| --- | --- | --- | --- | --- | --- |
| Uniform | 0.01 | 472 | 0.0002320 | 0.0002360 | 0.0002157 to 0.0002583 |
| Uniform | 0.05 | 11,338 | 0.0056667 | 0.0056690 | 0.0055659 to 0.0057740 |
| Uniform | 0.10 | 43,698 | 0.0220000 | 0.0218490 | 0.0216473 to 0.0220525 |
| Uniform | 0.25 | 250,989 | 0.1250000 | 0.1254945 | 0.1250361 to 0.1259543 |
| Biased | 0.01 | 478 | 0.0002643 | 0.0002390 | 0.0002185 to 0.0002614 |
| Biased | 0.05 | 12,707 | 0.0064425 | 0.0063535 | 0.0062443 to 0.0064646 |
| Biased | 0.10 | 49,957 | 0.0249400 | 0.0249785 | 0.0247631 to 0.0251957 |
| Biased | 0.25 | 281,103 | 0.1403125 | 0.1405515 | 0.1400705 to 0.1410339 |
| One alternative | 0.01 | 612 | 0.0002980 | 0.0003060 | 0.0002827 to 0.0003312 |
| One alternative | 0.05 | 14,408 | 0.0072500 | 0.0072040 | 0.0070877 to 0.0073222 |
| One alternative | 0.10 | 56,329 | 0.0280000 | 0.0281645 | 0.0279361 to 0.0283947 |
| One alternative | 0.25 | 312,450 | 0.1562500 | 0.1562250 | 0.1557225 to 0.1567288 |

Note: $\pi=(1/3,1/3,1/3)$ and $S_{2}=1/3$. Biased: $\pi=(0.8,0.1,0.1)$ and $S_{2}=0.66$. One alternative: $\pi=(1,0,0)$ and $S_{2}=1$. The two individual intervals not containing analytical value are the uniform setting at $p=0.25$ and the biased setting at $p=0.01$.
